# Exploring shared biomarkers and shared pathways in insomnia and atherosclerosis using integrated bioinformatics analysis

**DOI:** 10.3389/fnmol.2024.1477903

**Published:** 2024-10-08

**Authors:** Qichong Yang, Juncheng Liu, Tingting Zhang, Tingting Zhu, Siyu Yao, Rongzi Wang, Wenjuan Wang, Haliminai Dilimulati, Junbo Ge, Songtao An

**Affiliations:** ^1^Central China Fuwai Hospital of Zhengzhou University, Fuwai Central China Cardiovascular Hospital, Zhengzhou, Henan, China; ^2^Key Laboratory of Cardiac Regenerative Medicine, National Health Commission, Central China Subcenter of National Center for Cardiovascular Diseases, Henan Cardiovascular Disease Center, Zhengzhou, Henan, China; ^3^Henan Province People's Hospital, People's Hospital of Zhengzhou University, Zhengzhou, Henan, China; ^4^Center for Clinical Single-Cell Biomedicine, Henan Province People’s Hospital, People's Hospital of Zhengzhou University, Zhengzhou, Henan, China; ^5^Department of Cardiopulmonary Functions Test, Henan Province People’s Hospital, People's Hospital of Henan University, Zhengzhou, Henan, China; ^6^Department of Cardiology, Zhongshan Hospital, Fudan University, Shanghai, China

**Keywords:** insomnia, atherosclerosis, biomarkers, inflammatory response, immune cell infiltration, single-cell sequencing analysis

## Abstract

**Background:**

Insomnia (ISM) is one of the non-traditional drivers of atherosclerosis (AS) and an important risk factor for AS-related cardiovascular disease. Our study aimed to explore the shared pathways and diagnostic biomarkers of ISM-related AS using integrated bioinformatics analysis.

**Methods:**

We download the datasets from the Gene Expression Omnibus database and the GeneCards database. Weighted gene co-expression network analysis and gene differential expression analysis were applied to screen the AS-related gene set. The shared genes of ISM and AS were obtained by intersecting with ISM-related genes. Subsequently, candidate diagnostic biomarkers were identified by constructing protein–protein interaction networks and machine learning algorithms, and a nomogram was constructed. Moreover, to explore potential mechanisms, a comprehensive analysis of shared genes was carried out, including enrichment analysis, protein interactions, immune cell infiltration, and single-cell sequencing analysis.

**Results:**

We successfully screened 61 genes shared by ISM and AS, of which 3 genes (*IL10RA*, *CCR1*, and *SPI1*) were identified as diagnostic biomarkers. A nomogram with excellent predictive value was constructed (the area under curve of the model constructed by the biomarkers was 0.931, and the validation set was 0.745). In addition, the shared genes were mainly enriched in immune and inflammatory response regulation pathways. The biomarkers were associated with a variety of immune cells, especially myeloid immune cells.

**Conclusion:**

We constructed a diagnostic nomogram based on *IL10RA*, *CCR1*, and *SPI1* and explored the inflammatory-immune mechanisms, which indicated new insights for early diagnosis and treatment of ISM-related AS.

## 1 Introduction

Insomnia (ISM) is a most common sleep disorder all over the world, which seriously affects people’s quality of life and may even endanger health. Its most common symptoms are difficulty initiating and maintaining nocturnal sleep, decreased sleep quality, and reduced sleep duration (Sutton, 2021). As the pace of modern life increases, the incidence of ISM has increased, which often occurs simultaneously with obesity, diabetes mellitus, cardiovascular disease, etc. (Taylor et al., 2007; Madari et al., 2021). Some survey studies found that the incidence and the death risk of cardiovascular disease are significantly increased in the ISM patient population (Daghlas et al., 2019; Svensson et al., 2021). However, the pathogenesis is poorly understood.

Atherosclerosis (AS), the most common pathological basis of cardiovascular disease, is a chronic inflammatory vascular lesion with multifactorial associations (Libby, 2021). Several studies have indicated that short sleep duration and poor sleep quality are associated with enhanced arterial stiffness and AS development (Pan et al., 2022). Compared to subjects with 7–8 h of high-quality sleep, the risk of developing AS was found to be increased by 27% in those with less than 6 h of sleep and by 34% in subjects with fragmented sleep (Domínguez et al., 2019). It may be related to the inflammatory response evoked by ISM. It has been found that sleep deprivation increases serum levels of prostaglandin D2, induces accumulation of circulating neutrophils and cytokine storms, and causes multi-organ dysfunction (Sang et al., 2023). Chronic sleep loss initiates a systemic inflammatory response and produces significant increases in plasma levels of C-reactive protein, tumor necrosis factor-*α* receptor 1, and interleukin-6 (IL-6) (Shearer et al., 2001; Meier-Ewert et al., 2004). Moreover, excessive inflammation by ISM disrupts the normal rhythms of the hypothalamic–pituitary–adrenal axis, causes disorders of glucolipid metabolism, and raises the risk of diabetes and hyperlipidemia (Lim, 2019; Zhang et al., 2024). These are key risk factors for the development of AS. However, in the initial period of AS, patients normally have no obvious symptoms, which greatly challenges the diagnosis and treatment of AS. Therefore, finding more specific diagnostic markers for ISM patients with AS, and carrying out earlier treatment interventions is of great clinical significance.

In this study, based on ISM and AS gene expression data published in the Gene Expression Omnibus (GEO) and the GeneCards databases, we used a systems bioinformatics approach to explore shared gene pathways and diagnostic markers between ISM and AS, with the aim of identifying new potential diagnostic and therapeutic strategies for AS patients secondary to ISM.

## 2 Methods

### 2.1 Data collection and processing

**Figure 1:**
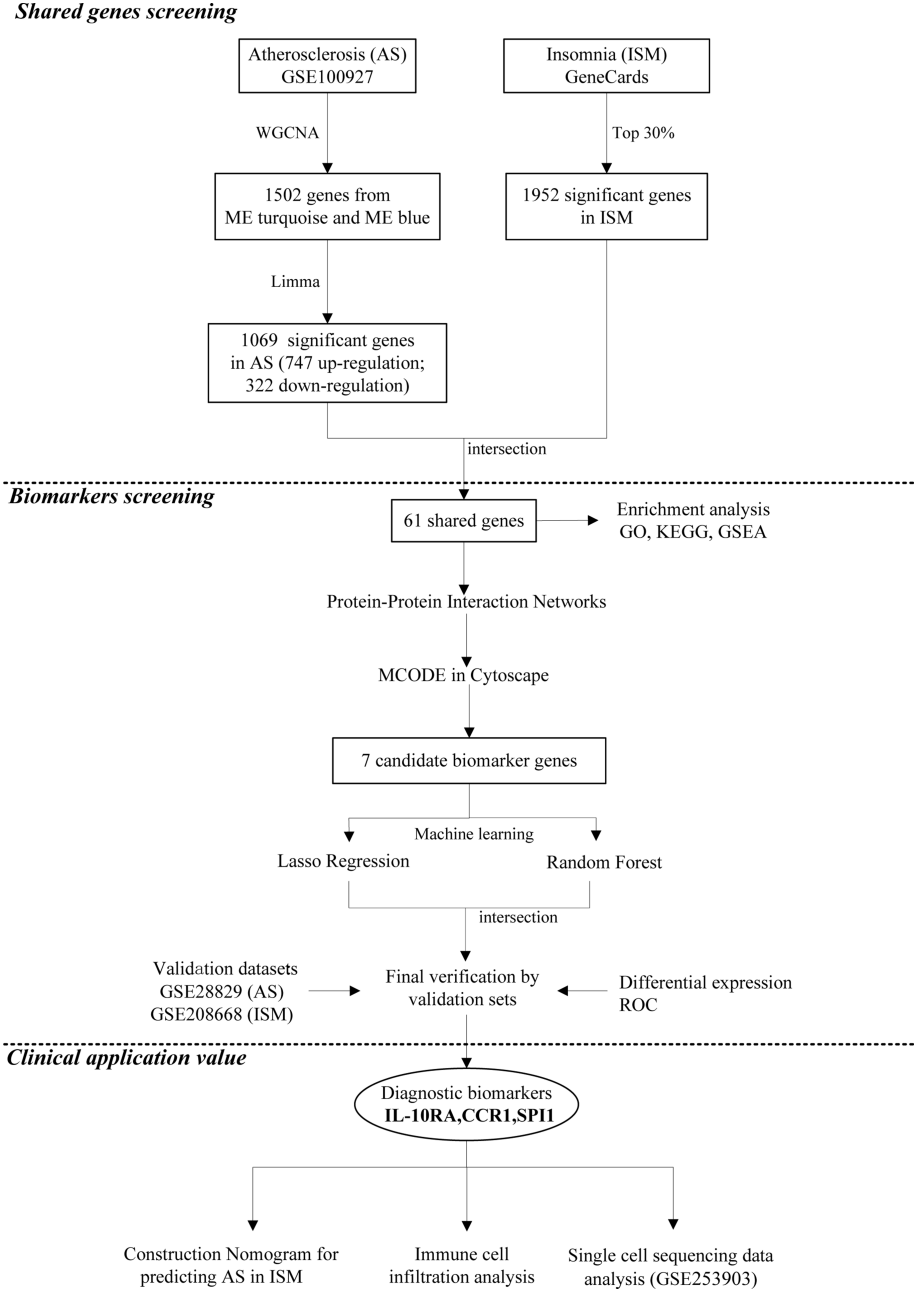
Flowchart depicting of the analysis process.

The data analysis process of our study is shown in Figure 1. Three microarray datasets (GSE100927, GSE28829, and GSE208668) were downloaded from the GEO database^1^ (Barrett et al., 2013) of the National Center for Biotechnology Information (NCBI) and a single-cell RNA sequencing (scRNA-seq) dataset (GSE253903). The basic information about the datasets is shown in Supplementary Table S1. The GSE100927 dataset contains 69 human arterial samples with AS lesions and 35 without (Steenman et al., 2018). The GSE28829 dataset contains 13 samples of early AS plaques and 16 samples of advanced (Döring et al., 2012). The GSE208668 contains 17 mononuclear cells of peripheral blood samples from patients with ISM and 25 from healthy individuals (Piber et al., 2022). In GSE208668, patients included were aged 60 years and above, with insomnia ≥3 times per week for >3 months, and had not suffered from other sleep disorders or chronic diseases. Removal of batch effects and normalization in microarray datasets was carried out by the “normalizeBetweenArrays” function of the R software “Limma” package (Supplementary Figure S1). The GSE253903 pre-processed by Cellranger (10X Genomics) contains 6 carotid AS plaques from symptomatic patients (Bashore et al., 2024). We searched and downloaded a collection of ISM-related genes in the GeneCards database^2^ (Safran et al., 2010) by the keyword “insomnia” and filtered the top 30% of genes (total 1,952) for subsequent analyses according to the “Relevance score.”

### 2.2 Weighted gene co-expression network analysis and identification of key module genes

WGCNA is a systems biology technique that reveals gene association patterns in different samples and identifies gene sets that have a significant correlation with phenotypes based on associations between gene sets and between gene sets and phenotypes. After normalization and removing the batch effect of the original datase, the identification was performed using the R software “WGCNA” package. The “goodSamplesGenes” function was used to check the unqualified genes and samples, and then the “pickSoftThreshold” function was used to pick the appropriate soft threshold power (*β* = 2) to construct the “unsigned” co-expression pattern. Finally, the gene modules were discovered using hierarchical clustering, and a cluster dendrogram was obtained using “plotDendroAndColors.” The module eigengenes (MEs) of different modules were obtained in the first principal component of the modules. Then, the module-trait correlation (Pearson correlation) was assessed based on the association between MEs and the clinical traits. The modules of highest relevance to the trait were selected, Module Membership (MM: the correlation coefficient of gene expression and MEs) and Gene Significance (GS: the correlation coefficient of gene expression and trait) coefficients were calculated (the thresholds: MM > 0.80 and GS > 0.50).

### 2.3 Analysis of differently expressed genes

The “Limma” package was used to identify the DEGs in the gene set obtained by WGCNA. The significance threshold was set at adjusted *p* < 0.05 and | log2 (fold change) | > 0.50. The filter results were visualized in a volcano plot and a heatmap by the “ggplot2” package and “pheatmap” package. The “VennDiagram” package was used to visualize shared genes between gene sets.

### 2.4 Functional enrichment analysis

The Gene Ontology (GO) term (BP, biological process; CC, cellular component; and MF, molecular function) enrichment analysis (significance *p* < 0.05 and *q* < 0.05), the Kyoto Encyclopedia of Genes and Genomes (KEGG) pathway enrichment analysis (*p* < 0.05 and *q* < 0.05), and the Gene Set Enrichment Analysis (GSEA) (*p* < 0.05 and *q* < 0.05) were carried out using the “clusterProfiler” package (version 4.10.0). The enrichment results were visualized using the “ggplot2” package and “enrichplot” package.

### 2.5 Construction of protein–protein interaction network

We constructed the PPI network on the STRING database (version 12.0) (Szklarczyk et al., 2023)^3^ (Minimum interaction requirement score: medium confidence = 0.400) and imported the results of the network into Cytoscape software (version 3.10.1) (Otasek et al., 2019) for visualization. To filter key sub-network modules from the PPI network, we utilized the Molecular Complex Detection (MCODE) plugin in Cytoscape (parameters: degree cutoff = 2, Node Score Cutoff = 0.2, K-Core = 2, max.Depth = 100). The genes in the key module were identified as the hub genes, which were used to further filter the diagnostic markers.

### 2.6 Machine learning algorithms

The least absolute shrinkage and selection operator (LASSO) regression model (a logistic regression method for filtering variables to enhance predictive performance) was constructed based on the “glmnet” package, and the minimum target covariate mean (lambda.min) was determined to filter the candidate biomarkers. The random forest (RF) model was constructed by the “randomForest” package, and the “MeanDecreaseAccuracy” and “MeanDecreaseGini” scores were calculated as the importance scores of the candidate biomarkers. Finally, the shared hub genes screened by the two models were used as candidate biomarkers.

### 2.7 Receiver operating characteristics curve and nomogram construction

Using the “ggpubr” package, the expression of candidate biomarkers in the GSE208668 and GSE28829 validation sets was compared and visualized in the box diagram. Using the “pROC” package, the diagnostic value of the diagnostic biomarkers was assessed by constructing the ROC, calculating the area under the curve (AUC), and calculating the 95% confidence interval. Nomogram was constructed using the R software “rms” package and its diagnostic efficiency was assessed by calculating the AUC.

### 2.8 Immune cell infiltration analysis

Cell-type identification by estimating relative subsets of RNA transcript (CIBERSORT) is a computational method used to translate a normally differentiated gene expression matrix into an infiltrating immune cell proportion. Using the CIBERSORT.R script (Newman et al., 2015), the relative proportions of the infiltration of 22 immune cells in each sample were calculated and depicted in the bar diagram. The comparison of the differential expression of each immune cell between the AS group and the controls is shown by the box diagram. The correlation between diagnostic biomarkers and immune cells (spearman correlation) was calculated using the “corrplot” package.

### 2.9 Single-cell RNA sequencing data analysis

After downloading the dataset (GSE253903), it was analyzed by the “Seurat” package (version 5.0.1). Quality control of the data was carried: gene counts per cell in the range of 200–2,500 and a percentage of mitochondrial genes less than 5%. Next, the data were normalized using the NormalizeData function, the first 2000 highly variable genes were selected using the “vst” method in the FindVariableFeatures function, the data were scaled using the ScaleData function, and the clustering analysis was performed using the RunPCA function for cluster analysis. We utilized the “harmony” package (version 1.2.0) to eliminate batch effects. Clustering and dimensionality reduction were carried out using FindNeighbors, FindClusters, and RunTSNE functions (dim = 1:20, resolution = 0.5) (Supplementary Figures S2B,C). Subsequently, the cell clusters were visualized using the DimPlot function for visualization. The FindAllMarkers function was utilized to identify the top three marker genes for per cluster. Cell cluster annotation was performed using a strategy of automatic annotation combined with manual correction. Firstly, cell types were annotated using the “SingleR” package (version 2.4.1), and HumanPrimaryCellpronasData downloaded from the “celldex” package (version 1.12.0) was used as the reference datasets. Then, cell types were identified in the CellMarker 2.0 database (Hu et al., 2023)^4^ using the marker genes for each cell cluster. The cell types of each cluster were visualized using DimPlot and DotPlot functions. We utilized the FeaturePlot and VlnPlot functions for visualization to clarify the cellular localization of diagnostic biomarkers.

### 2.10 Statistical analysis

R software (version 4.3.2) (R Core Team, 2023)^5^ and RStudio software (version 2023.12.1)^6^ were used for data analysis and drawing. The Wilcoxon test was used to compare the differences between the two groups in the test sets. Statistical significance was inferred when *p*-value < 0.05.

## 3 Results

### 3.1 Identification of the AS-related genes

**Figure 2:**
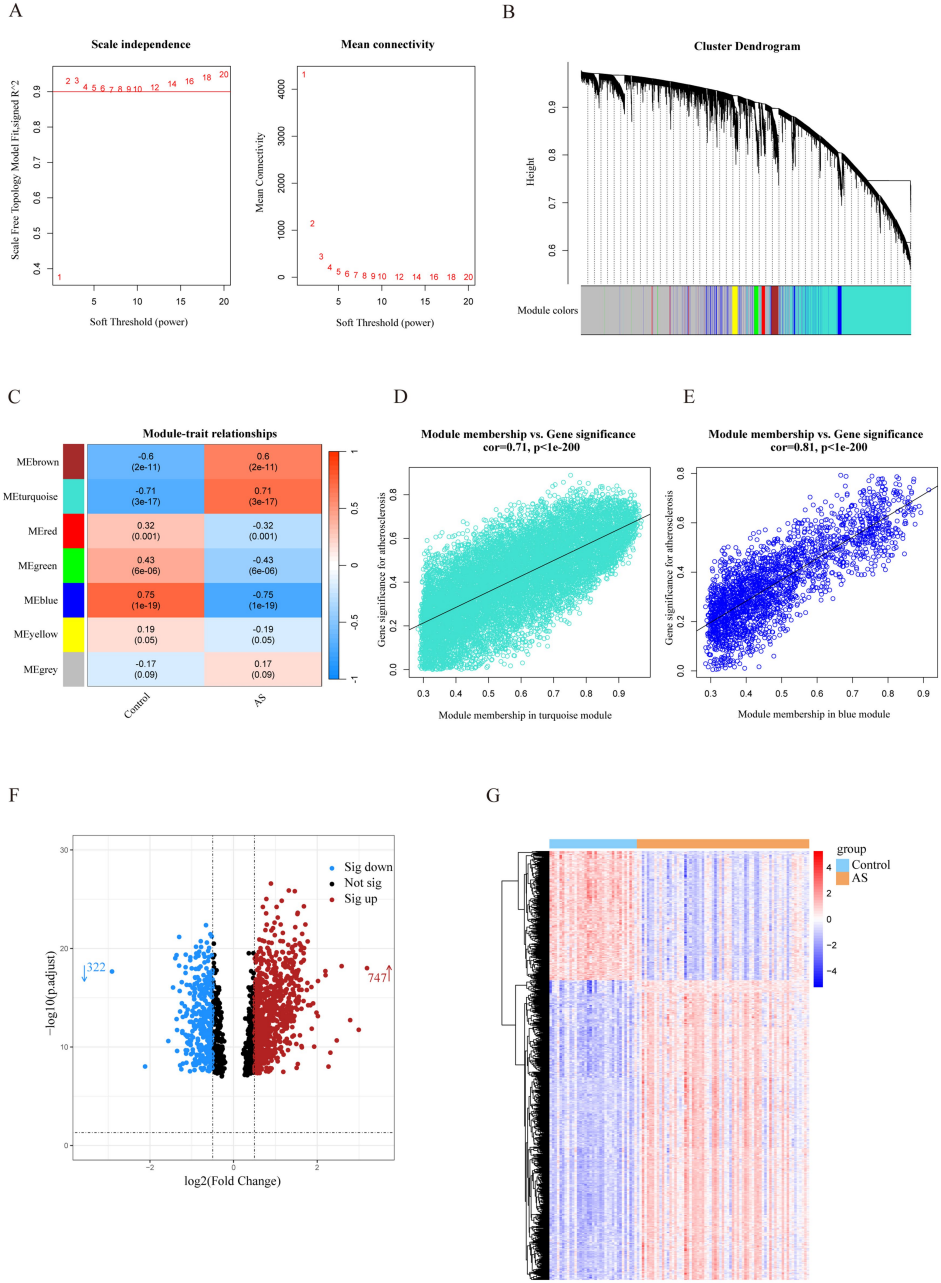
Identification of AS-related genes by WGCNA combined with the Limma strategy. **(A)** Soft thresholding power (*β*) selection via scale independence and average connectivity. **(B)** Cluster dendrogram of gene clusters or modules associated with atherosclerosis. **(C)** The heatmap depicting correlation between modules and clinical traits in AS. The top number represents the correlation coefficient, and the bottom number represents the *p* value in the squares. **(D,E)** The correlation between module membership and gene significance in AS regarding the most positively (turquoise module) and negatively (blue module) correlated modules. **(F)** The volcano plot of all DEGs in key modules of AS, with red and blue dots referring to significant up- and down-regulated DEGs. **(G)** The heatmap of the significant up- and down-regulated DEGs. Red and blue grids represent significant up- and down-regulated DEGs. ME, module eigenvectors; cor, correlation; WGCNA, weighted gene co-expression network analysis; AS, atherosclerosis.

The most strongly related gene modules and gene sets in the AS samples were identified by WGCNA. The most acceptable soft thresholding power (*β* = 2, *R*^2^ = 0.930) was chosen based on scale independence and average connectivity (Figure 2A). A total of 7 gene modules were generated in the soft threshold, and the cluster tree diagram of the modules was constructed (Figure 2B). Then, we evaluated the correlation between gene modules and AS (Figure 2C). The results showed the strongest positive correlation between the turquoise module (ME turquoise) and AS (correlation coefficient *r* = 0.71, *p* = 3e-17), whereas the blue module (ME blue) had the strongest negative correlation (*r* = −0.75, *p* = 1e-19). In addition, we found a strong correlation between MM and GS in the turquoise module (*r* = 0.71, *p* < 1e-200) and the blue module (*r* = 0.81, *p* < 1e-200) (Figures 2D,E), which indicated a significant correlation between the module genes and AS. Therefore, we filtered the total of 1,502 genes (MM > 0.8 and GS > 0.5) from the two key modules for subsequent analysis.

To filter out genes with a stronger correlation with AS, we analyzed the differential expression of the key module genes. A total of 1,069 DEGs were identified, of which 747 were up-regulated and 322 were down-regulated (Figure 2F). The heatmap demonstrated that the expression of these genes was significantly different in the AS and control groups (Figure 2G). Therefore, the DEGs were identified as a set of AS-related genes.

### 3.2 Identification of the ISM and AS shared genes and shared pathways

**Figure 3:**
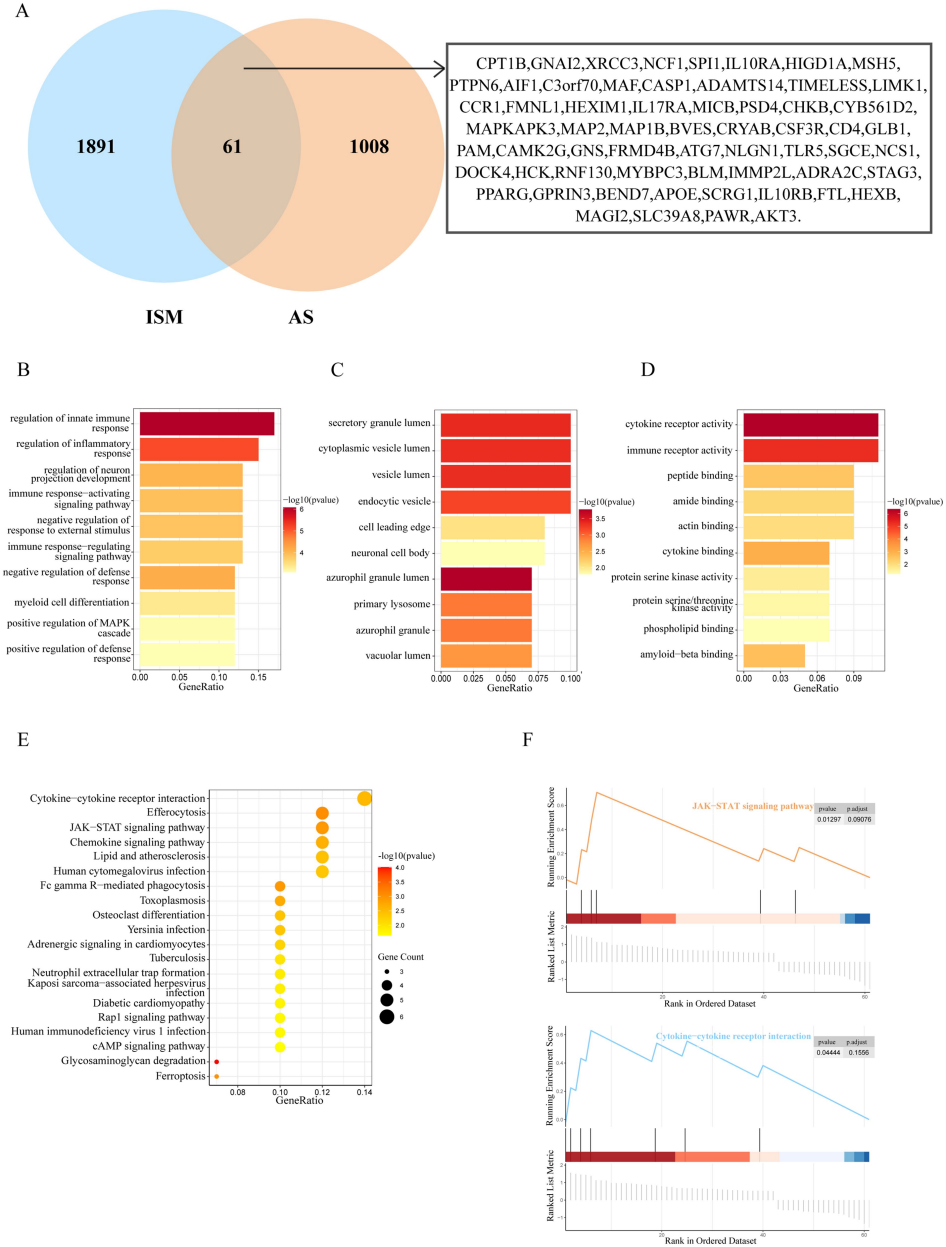
Identification and enrichment analysis of ISM and AS shared genes. **(A)** The venn diagram depicting the 61 shared genes of ISM and AS. The shared genes were displayed on the right labels. **(B–D)** GO analysis of shared genes (**B**, biological process; **C**, cellular component; **D**, molecular function). The top 10 enriched GO categories are visualized via a bar diagram. The X and Y axes represent the gene ratio and different ontologies. The color denotes the *p*-value. **(E)** KEGG analysis of shared genes. The top 20 enriched KEGG categories are visualized via bubble diagram. The X and Y axes represent the gene ratio and different ontologies. The circle size denotes gene count, whereas the color, *p*-value. **(F)** GSEA analysis of shared genes. Only two enriched GSEA categories of a *p*-value < 0.05 are visualized. GO, Gene Ontology; KEGG, Kyoto Encyclopedia of Genes and Genomes; GSEA, Gene Set Enrichment Analysis.

After taking the intersection of the set of ISM-related genes searched from the GeneCards database with the set of AS-related genes, 61 genes were identified as the shared genes of ISM and AS (Figure 3A). Enrichment analyses of shared genes included GO, KEGG, and GSEA. The GO analysis revealed that the shared genes were majorly enriched for the following functions: (1) biological processes, regulation of innate immune response, regulation of inflammatory response, regulation of neuron projection development, and immune response-activating signaling pathway; (2) cellular component, secretory granule lumen, cytoplasmic vesicle lumen, vesicle lumen, and endocytic vesicle; (3) molecular function, cytokine receptor activity, immune receptor activity, peptide binding, and amide binding (Figures 3B–D). The KEGG analysis revealed that the shared genes were majorly enriched in the following pathways: cytokine-cytokine receptor interaction, efferocytosis, Janus kinase-signal transducer and activator of transcription (JAK–STAT) signaling pathway, chemokine signaling pathway, and lipid and atherosclerosis (Figure 3E). The GSEA revealed that the shared genes were significantly enriched in JAK–STAT signaling pathway and cytokine-cytokine receptor interaction (Figure 3F). These results indicate that inflammation and immune responses may have a vital effect on the development of AS in ISM patients.

### 3.3 Construction of PPI networks and identification of the hub genes

**Figure 4:**
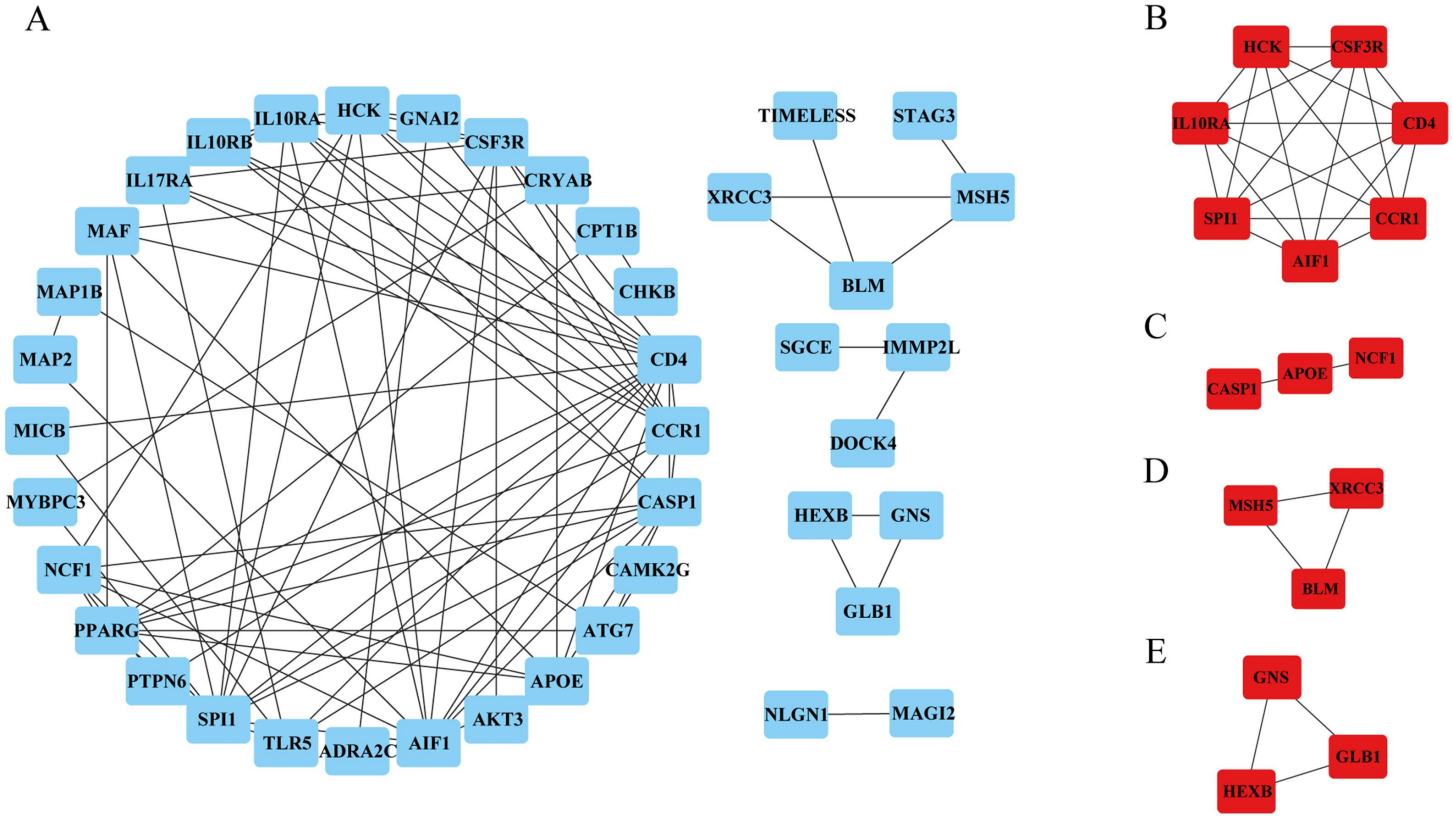
Protein–protein interaction (PPI) network construction and hub genes selection. **(A)** The PPI network shows the total interactions of the shared genes of ISM and AS. **(B–E)** Module 1–4. Filtering key interaction modules from PPI networks via the MOCDE algorithm. MOCDE, molecular complex detection.

To identify the hub genes of ISM-related AS, we uploaded the above shared genes to the STRING database, constructed a PPI network, and visualized it in Cytoscape software. After removing the genes that did not interact with other shared genes, a PPI network containing 41 nodes and 89 edges was constructed (Figure 4A). Then, 4 key modules were identified from the PPI network using the MCODE plugin in Cytoscape (Figures 4B–E). Briefly, module 1 was comprised of 7 genes, including *CD4*, *AIF1*, *IL10RA*, *CCR1*, *HCK*, *SPI1*, and *CSF3R*. Module 2 comprised 3 genes, including *CASP1*, *APOE*, and *NCF1*. Module 3 comprised 3 genes, including *MSH5*, *XRCC3*, and *BLM*. Module 4 comprised 3 genes, including *GNS*, *GLB1*, and *HEXB*. Obviously, module 1, containing 7 nodes and 21 edges, had the most complex interrelationships. Thus, it was identified as the hub module, and the 7 genes in module 1 were identified as the hub genes. The hub genes have complex interactions with each other, which may have a key role in ISM-accelerated AS.

### 3.4 Selection of candidate diagnostic biomarkers for ISM-related AS using machine learning

**Figure 5:**
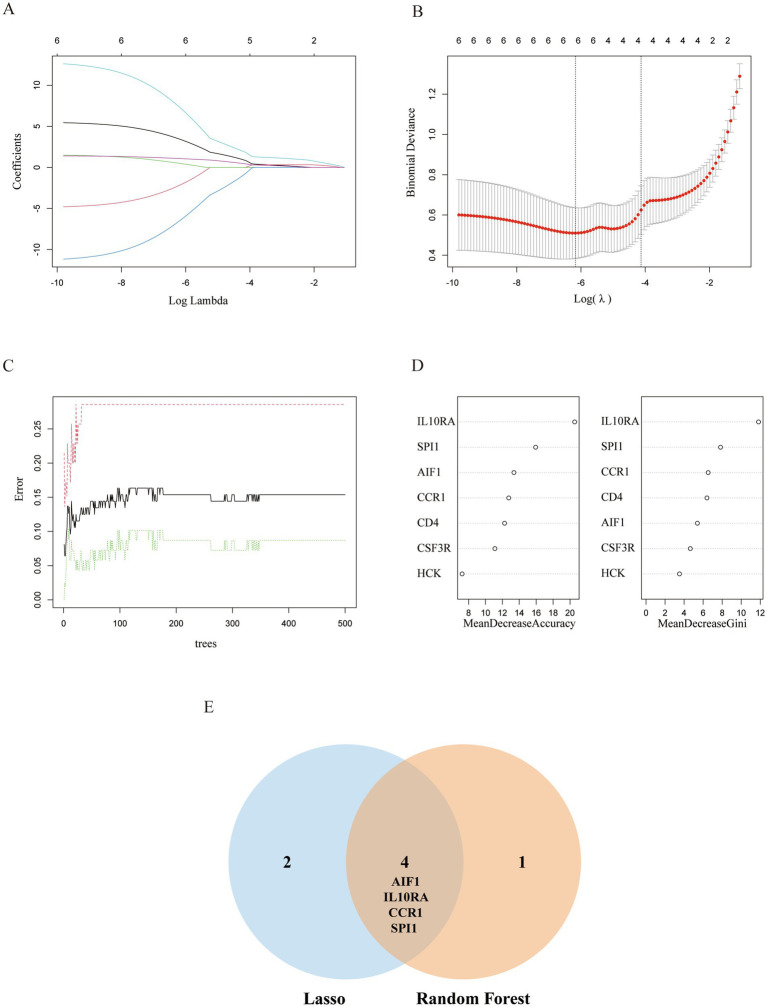
Identification of candidate diagnostic biomarkers for ISM-related AS by machine learning methods. **(A,B)** LASSO regression analysis was applied to screen diagnostic biomarkers based on the 7 hub genes. The hub genes with the minimum binominal deviance were identified as the most suitable candidate genes. **(C,D)** RF algorithm used to rank the importance of 7 Hub genes. The top 5 genes in the importance ranking graph **(D)** were identified as the most suitable candidate genes. **(E)** The Venn diagram depicting common candidate diagnostic markers of LASSO and RF. ISM, insomnia; AS, atherosclerosis; LASSO, least absolute shrinkage and selection operator; RF, random forest; *AIF1*, allograft inflammatory factor 1; *IL10RA*, interleukin 10 receptor subunit alpha; *CCR1*, C-C motif chemokine receptor 1; *SPI1*, Spi-1 proto-oncogene.

The hub genes of ISM and AS may contribute to the diagnosis of ISM-related AS patients; therefore, we further selected the diagnostic biomarkers using machine learning. The LASSO regression analysis identified the 6 genes with the lowest binomial deviation among the hub genes with the best fit to the regression model (Figures 5A,B). In addition, we screened the hub genes using the RF algorithm and selected the genes with the top 5 of “MeanDecreaseAccuracy” and “MeanDecreaseGini” scores (Figures 5C,D). After intersecting the results of the above two machine learning algorithms, the 4 candidate diagnostic biomarker genes were selected, including *AIF1*, *IL10RA*, *CCR1*, and *SPI1* (Figure 5E).

### 3.5 Validation of expression levels and assessment of diagnostic value of candidate diagnostic biomarkers and construction of the nomogram

**Figure 6:**
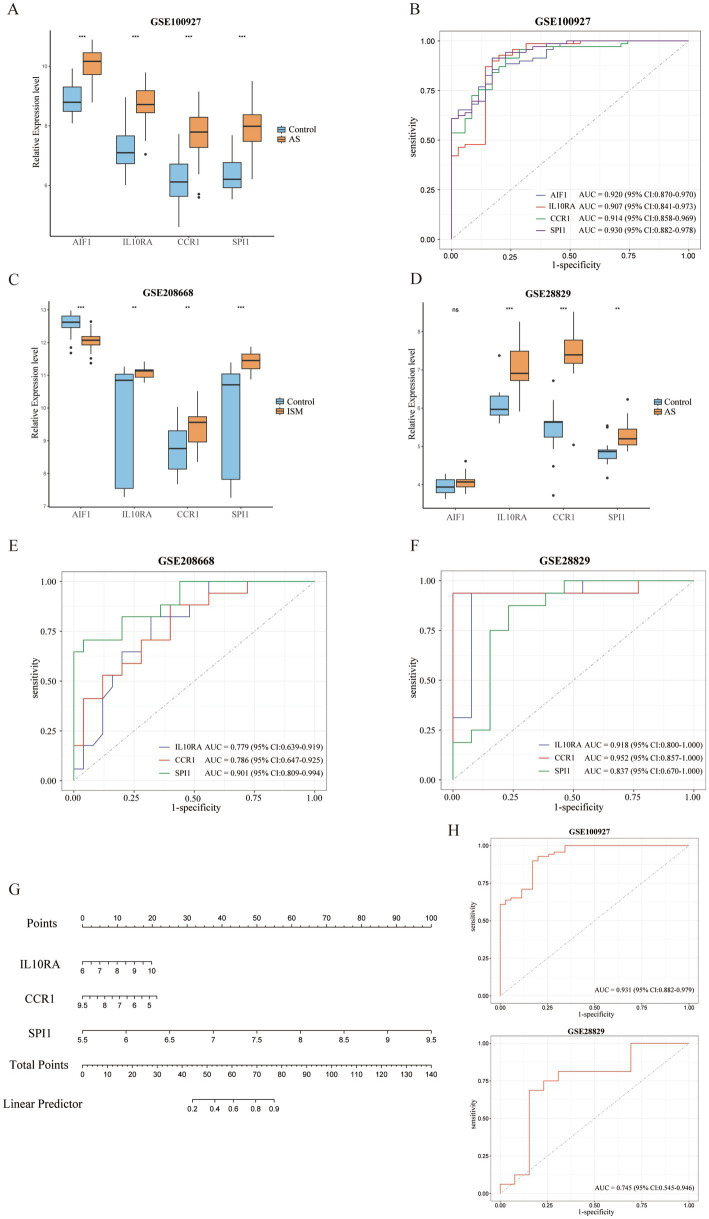
Validation of expression levels and assessment of the diagnostic value of candidate diagnostic biomarkers and nomogram establishment. **(A)** Expression comparison of 4 candidate biomarkers (*AIF1*, *IL10RA*, *CCR1*, and *SPI1*) in GSE100927. **(B)** The ROC curves of the candidate biomarkers in GSE100927. The AUCs and 95% CIs are displayed at the bottom. (C-D) Expression comparison of the candidate biomarkers in GSE208668 and GSE28829. **(E,F)** The ROC curves of 3 diagnostic biomarkers (*IL10RA*, *CCR1*, and *SPI1*) in GSE208668 and GSE28829. The AUCs and 95% CIs are displayed at the bottom. **(G)** The nomogram was established based on the diagnostic biomarkers. Each of the diagnostic biomarkers corresponds to a score. The total score of the biomarkers is used to predict the risk of AS in a population with ISM. **(H)** The ROC curve of the nomogram in AS datasets (GSE100927 and GSE28829). The AUC and 95% CI are displayed at the bottom. ISM, insomnia; AS, atherosclerosis; ROC, receiver operating characteristics curve; AUC, area under the curve; CI, confidence interval; *AIF1*, allograft inflammatory factor 1; *IL10RA*, interleukin 10 receptor subunit alpha; *CCR1*, C-C motif chemokine receptor 1; *SPI1*, Spi-1 proto-oncogene.

All 4 candidate diagnostic markers (*AIF1*, *IL10RA*, *CCR1*, and *SPI1*) were significantly up-regulated in the test set GSE100927 (AS), and the AUCs of the ROCs were over 0.9 (Figures 6A,B). However, in validation sets GSE208668 (ISM) and GSE28829 (AS), it was found that the expression trend of *AIF1* was different in the two diseases and the differential expression in GSE28829 was not statistically significant, while *IL10RA*, *CCR1*, and *SPI1* were up-regulated and the differences were statistically significant (Figures 6C,D). Then, the diagnostic value was assessed by constructing the ROCs and calculating the AUCs. Notably, *IL10RA*, *CCR1*, and *SPI1* had satisfactory diagnostic value: in GSE208668, *IL10RA*, AUC = 0.779, 95%CI: 0.639–0.919; *CCR1*, AUC = 0.786, 95%CI: 0.647–0.925; *SPI1*, AUC = 0.901, 95%CI: 0.809–0.994; in GSE28829, *IL10RA*, AUC = 0.918, 95%CI: 0.800–1.000; *CCR1*, AUC = 0.952, 95%CI: 0.857–1.000; *SPI1*, AUC = 0.837, 95%CI: 0.670–1.000 (Figures 6E,F). Therefore, we finally identified *IL10RA*, *CCR1*, and *SPI1* as diagnostic biomarkers. Moreover, to enhance the feasibility of clinical application, the 3 diagnostic markers were utilized to construct the nomogram (Figure 6G). In the nomogram, the expression level of each gene was scored accordingly, and the total score was used to predict the probability of AS. Finally, in the test and validation datasets of AS, the AUC of the nomogram was 0.931 (95% CI: 0.882–0.979) and 0.745 (95% CI: 0.545–0.946), indicating satisfactory diagnostic efficacy (Figure 6H).

### 3.6 Immune cell infiltration analysis

**Figure 7:**
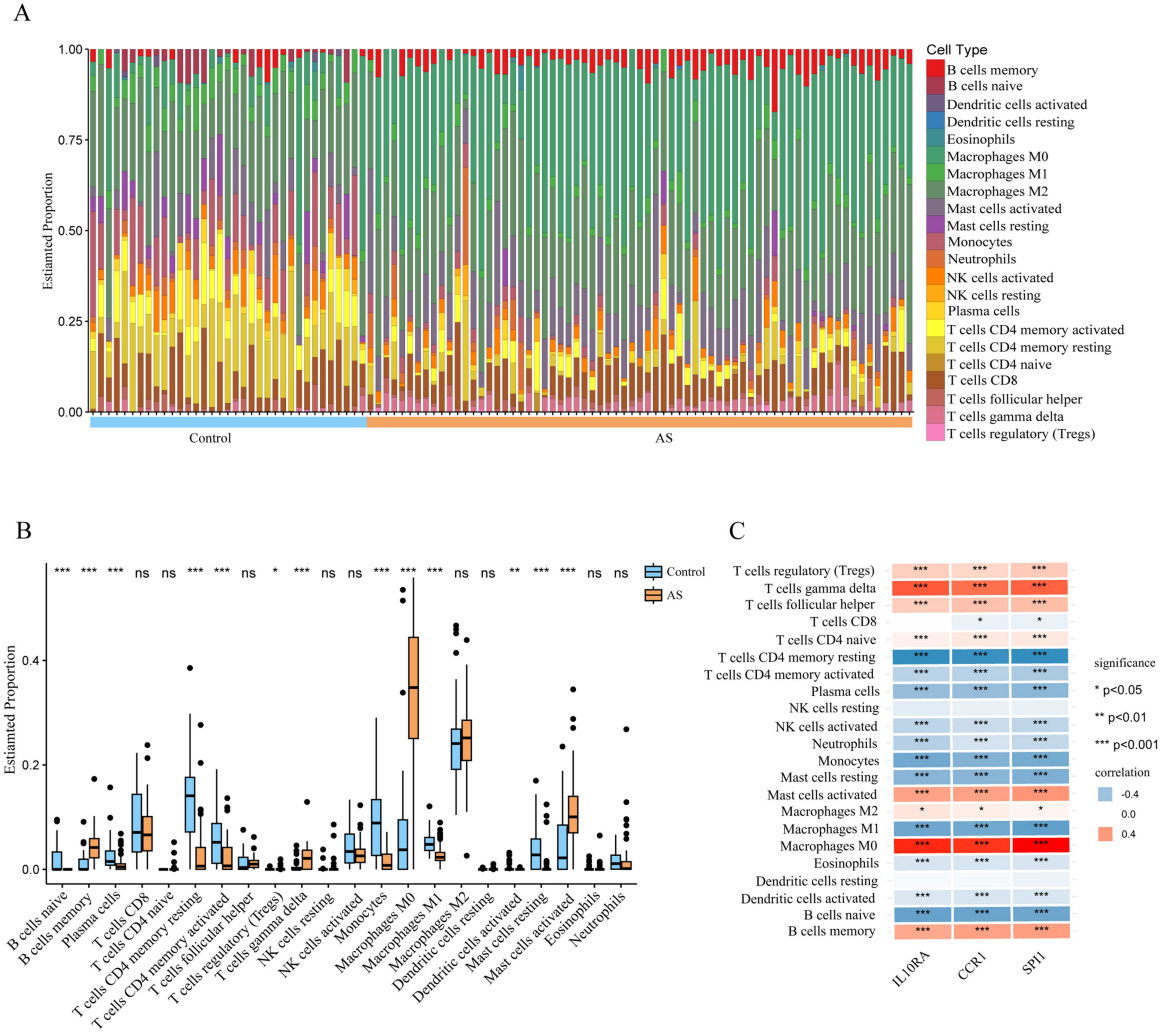
Immune cell infiltration in atherosclerotic plaque from patients with AS. **(A)** The relative proportion of 22 types of immune cells in AS and control samples is shown as a column proportion diagram. **(B)** Box-plot of the proportion of 22 types of immune cells. *p < 0.05, **p < 0.01, ***p < 0.001, ns *p* ≥ 0.05. **(C)** The correlation between 3 diagnostic biomarkers (*IL10RA*, *CCR1*, and *SPI1*) and the proportion of 22-type immune cells in AS is displayed as a heatmap. AS, atherosclerosis; *IL10RA*, interleukin 10 receptor subunit alpha; *CCR1*, C-C motif chemokine receptor 1; *SPI1*, Spi-1 proto-oncogene.

Since the functional and pathway enrichment results of the shared genes indicated a potential link between inflammatory-immune with ISM-related AS, we analyzed the characteristics of 22 types of immune cells in AS samples using the CIBERSORT algorithm (Figure 7A). In particular, the proportions of B cells memory, T cells regulatory (Tregs), T cells gamma delta, Macrophages M0, and Mast cells activated were significantly increased in AS samples (all *p* < 0.05), while the proportions of B cells naive, plasma cells, T cells CD4 memory resting, T cells CD4 memory activated, monocytes, macrophages M1, dendritic cells activated, and mast cells resting were significantly decreased (all *p* < 0.05) (Figure 7B). Moreover, we analyzed the correlation between gene expression of the three diagnostic biomarkers and the proportions of immune cell infiltration and found that the expression of *IL10RA*, *CCR1*, and *SPI1* were all significantly positively correlated with the proportions of macrophages M0, T cells gamma delta, and mast cells activated (all *p* < 0.05) and negatively correlated with the proportions of T cells CD4 memory resting, monocytes, and macrophages M1 (all *p* < 0.05) (Figure 7C). The diagnostic biomarker genes had the highest correlation coefficients with macrophages M0 proportion (*IL10RA*, 0.758; *CCR1*, 0.695; *SPI1*, 0.774). These results suggest that there may be important interactions between diagnostic biomarkers and various immune cells.

### 3.7 Identification of *IL10RA*, *CCR1*, and *SPI1* expression in AS plaques based on the scRNA-seq data

**Figure 8:**
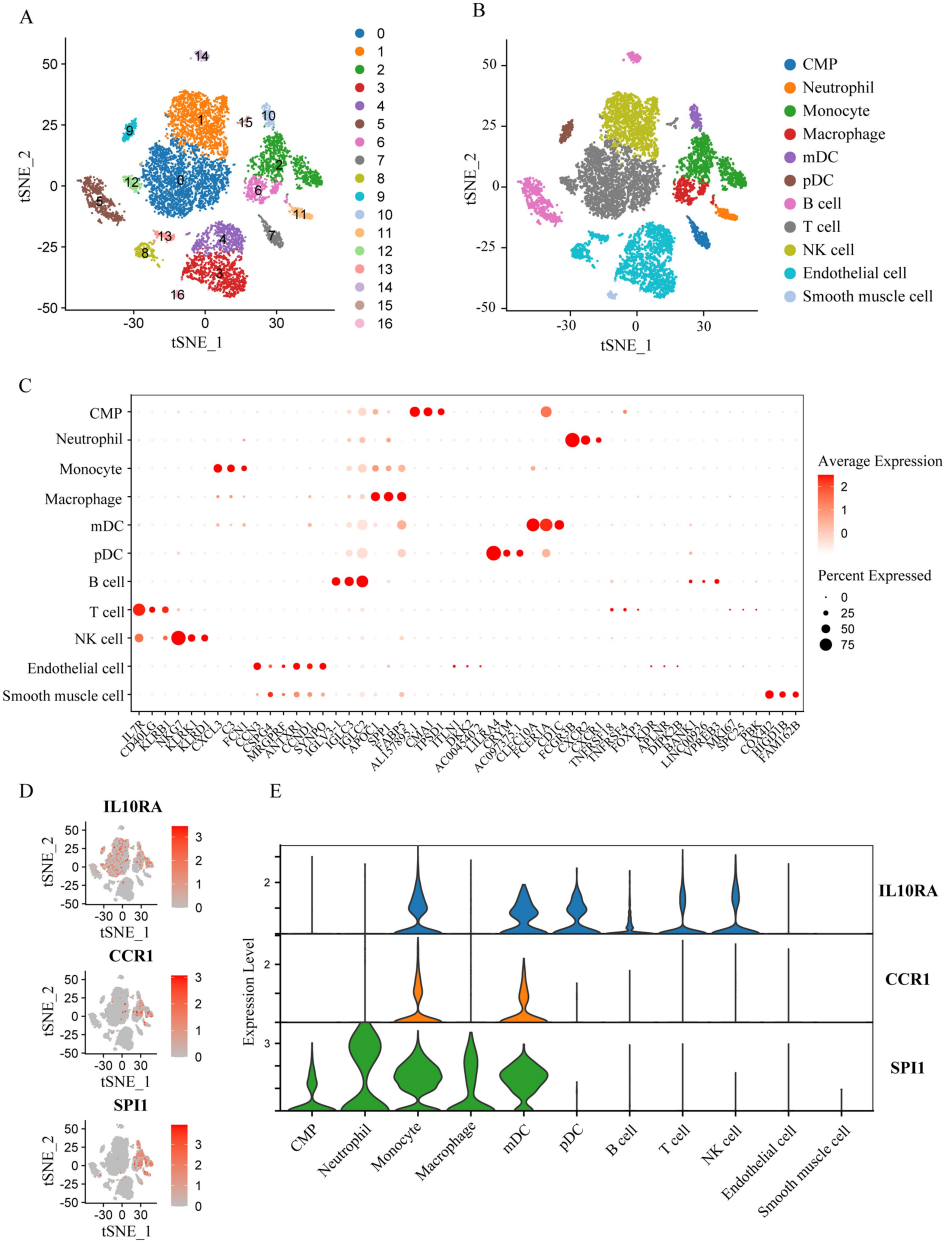
Identification of *IL10RA*, *CCR1*, and *SPI1* expression in AS plaque cells based on single-cell sequencing data (GSE253903). **(A)** The tSNE visualization of clustering revealing 17 cell clusters. **(B)** The tSNE visualization of the identification of 11 cell types from the 17 cell clusters. Cluster identities: 7, CMP; 11, Neutrophil; 2, Monocyte; 6, Macrophage; 10, mDC; 9, pDC; 5,14, B cell; 0,12,15, T cell; 1, NK cell; 3,4,8,13, Endothelial cell; 16, Smooth muscle cell. **(C)** The bubble plot of the top 3 marker genes for each cell cluster. **(D)** The tSNE visualization of the 3 diagnostic biomarker (*IL10RA*, *CCR1*, and *SPI1*) expression in cell clusters. **(E)** The violin plot of the 3 diagnostic biomarker expression in cell clusters. *IL10RA*, interleukin 10 receptor subunit alpha; *CCR1*, C-C motif chemokine receptor 1; *SPI1*, Spi-1 proto-oncogene; AS, atherosclerosis; CMP, common myeloid progenitor; mDC, myeloid dendritic cell; pDC, plasmacytoid dendritic cell; NK cell, natural killer cell; tSNE, t-Distributed Stochastic Neighbor Embedding.

To more precisely describe the immune cell traits and identify the cell types with significant expression of *IL10RA*, *CCR1*, and *SPI1* in AS plaques, we carried out bioinformatics analysis of the scRNA-seq dataset (GSE253903). After quality control, screening, normalization, and removal of batch effects from the raw data (Supplementary Figure S2A), we performed dimensionality reduction and clustering analysis based on gene expression profiles, and finally obtained a total of 17 cell clusters (Figure 8A). Then, cell annotations identified 11 cell types: common myeloid progenitor (CMP), neutrophil, monocyte, macrophage, myeloid dendritic cell (mDC), plasmacytoid dendritic cell (pDC), B cell, T cell, natural killer (NK) cell, endothelial cell, and smooth muscle cell (Figures 8B,C). Cell expression analysis of the diagnostic marker genes revealed that *IL10RA* was mainly expressed in monocyte, mDC, pDC, B cell, T cell and NK cell, *CCR1* in Monocyte and mDC, and *SPI1* in CMP, neutrophil, monocyte, macrophage and mDC (Figures 8D,E). These results indicated that the diagnostic biomarker genes were predominantly expressed in immune cells in AS plaques, with *CCR1* and *SPI1* expression especially clustered in myeloid immune cells.

## 4 Discussion

Existing studies have shown a potential link between ISM and AS (Pan et al., 2022; Sigurdardottir et al., 2023). Insomnia challenges the body’s immune system leading to a prolonged state of abnormal inflammatory activation (Irwin et al., 2016; Sang et al., 2023), and chronic inflammation is a key factor in the development of AS (Lawler et al., 2021). Currently, the causal relationship between ISM and AS cannot be established. Therefore, identifying the common features of these two disorders could help to explore the causal relationship between them and develop new effective diagnostic and preventive strategies. In this study, we explored the common disease pathways and diagnostic markers involved in ISM and AS using bioinformatic analysis, and found that aberrant activation of inflammatory-immune pathways might be the potential mechanism for ISM-accelerated AS. More importantly, the 3 biomarkers closely related to inflammation and immunity (*IL10RA*, *CCR1*, and *SPI1*) were identified, and a nomogram was constructed, which demonstrated a satisfactory clinical predictive value. In addition, we found that the diagnostic biomarkers were strongly correlated with the infiltration of multiple immune cells and were predominantly expressed in immune cells in AS plaques, among which *CCR1* and *SPI1* were centrally expressed in myeloid immune cells. It provides insights to further explore the mechanism of ISM-accelerated AS.

As the signaling cascade response representative and one of the central cellular communication nodes, the JAK–STAT signaling pathway contains more than 50 cytokines and growth factors, such as interferon, interleukin, colony-stimulating factors, hormones, etc. (Hu et al., 2021). This pathway can be involved in immune regulation, cell proliferation, differentiation and apoptosis, hematopoietic and tumor proliferation, and has been associated with the development of a variety of diseases, including inflammatory diseases, immune diseases, cancers, and hematological disorders (Philips et al., 2022). Recently, it was shown that sleep deprivation in rats activated the JAK–STAT signaling pathway, causing an inflammatory response and atrophy of the rat biting muscle (Gomes Galvani et al., 2021). Conversely, interventions that inhibit this pathway improved the activated inflammatory response in animal models of obstructive sleep apnea (Hsiao et al., 2024). In addition, the JAK–STAT signaling pathway is closely related to AS. Interferon-*γ* activates the JAK–STAT signaling pathway by interacting with Janus kinase 1 (JAK1) and JAK2 to result in AS (Boshuizen and de Winther, 2015). Crucially, inhibitors of the JAK–STAT pathway ameliorate AS exacerbated by lipopolysaccharide, making it a potential therapeutic target (Hashimoto et al., 2020). Therefore, we believe that the JAK–STAT signaling pathway may be a key bridge linking ISM and AS.

The interleukin 10 receptor subunit alpha (*IL10RA*) encodes the protein that is the receptor of interleukin 10 (IL-10), which mediates the immunosuppressive signaling of IL-10, thereby inhibiting the synthesis of pro-inflammatory cytokines (Sabat et al., 2010). Several studies have found that poor sleep quality (Yang et al., 2023) and sleep deprivation (Zhai et al., 2021) were positively associated with the blood IL-10 concentration, and chronic circadian dysregulation also increased IL-10 (Wright et al., 2015). Similarly, *IL10RA* expression was upregulated in AS plaques of coronary and carotid arteries (Cagnin et al., 2009). Increasing the concentration of IL-10 *in vivo* by delivering IL-10 mRNA drugs to exert the anti-inflammatory effects of *IL10RA* may be a promising strategy for anti-AS therapy (Gao et al., 2023). C-C motif chemokine receptor 1 (*CCR1*) encodes a 7-transmembrane protein similar to the G protein-coupled receptor, which is expressed on a variety of immune cells and is involved in inflammatory signaling and leukocyte recruitment in inflammatory responses (Tsou et al., 1998; Horuk, 2001). There is limited evidence for a relationship between *CCR1* and ISM, but genetic studies have found that a single-nucleotide polymorphism in *CCR1* (rs3181077) was more prevalent in patients with early narcolepsy, which is a chronic neurologic sleep disorder (Ouyang et al., 2020). However, *CCR1* in monocytes stimulated by platelet factor 4 (Fox et al., 2018) or C-C motif chemokine ligand 5 (Jehle et al., 2018) causes monocytes to migrate and recruit on the vascular endothelium, promoting the vascular inflammatory response. This is critical in early plaque formation in AS. Therapeutic strategies targeting *CCR1* and its associated chemokine pathways are also extremely potential in cardiovascular disease treatment (Márquez et al., 2021). Spi-1 proto-oncogene (*SPI1*) encodes an ETS structural domain transcription factor, which regulates hematopoietic cell fate by directly controlling gene expression through the binding of gene regulatory elements, and is required for the later stages of myeloid and B-lymphocyte development (Pham et al., 2013). *SPI1* exhibits a wide range of functional regulatory roles and has been associated with a variety of immune and inflammatory diseases (Fan et al., 2023; Liu et al., 2023; Xie et al., 2023). Remarkably, sufficient sleep also regulates hematopoiesis and prevents the development of AS (McAlpine et al., 2019). In some studies, *SPI1* has been predicted to be a potential transcription factor for AS-related genes, intervening in AS by regulating gene expression (Cui et al., 2023; Zhang et al., 2023). In recent years, with the proposed atherogenesis theories of “clonal hematopoiesis” (Polizio et al., 2023) and “smooth muscle cell tumor-like changes” (Pan et al., 2024), the function and mechanism of *SPI1* in the formation of AS deserves more intensive studies.

Mature atherosclerotic plaques contain a variety of immune cell types, among which myeloid cells (including monocytes, neutrophils, macrophages, mDCs, etc.) are key participants in atherosclerosis, and the alteration of the balance of pro- and anti-inflammatory myeloid cells in the arterial vessel wall is strongly related to the development of AS (Chistiakov et al., 2019; Vallejo et al., 2021). Hyperactivation of myeloid cells leads to reactivation of T cells and the production of large amounts of proatherosclerotic cytokines (Peshkova et al., 2017). It was found that knocking out certain specific genes in mouse myeloid cells significantly limited the inflammatory response and reduced the development of AS (Doddapattar et al., 2022; Singla et al., 2022). Moreover, a mendelian randomization study found that immune cell characteristics of monocytes and mDCs were associated with an increased risk of insomnia (Han et al., 2024). Sleep loss affects the distribution of myeloid cell subsets and induces the development of inflammation and cell senescence (Liu et al., 2021). As such, it seems that immune cells, especially myeloid cells, may be an important mediator linking ISM and AS.

However, there are several limitations to our study. First, the diagnostic biomarkers we identified were derived from the analysis of arterial tissue samples, although they were mainly expressed in immune cells. For translation to clinical applications, expanded sample sizes and types are needed to further explore the expression of diagnostic markers in the various tissues, especially in blood samples. Second, although we suggested the potential pathways and validated the 3 diagnostic biomarkers, our study began with the analysis of the public data sets, and more basic and clinical experiments will be necessary to validate the results in the future.

## 5 Conclusion

Our study identified that the immune-inflammatory response has an important role in ISM-related AS. Three diagnostic biomarkers were identified by machine learning algorithms, and the nomogram was constructed to provide an early diagnosis of clinical ISM-associated AS in the clinic. In addition, the diagnostic biomarkers were strongly associated with the myeloid immune cells, suggesting potential therapeutic strategies.

## Data Availability

The original contributions presented in the study are included in the article/Supplementary material, further inquiries can be directed to the corresponding authors.

## Footnotes

^1^ https://www.ncbi.nlm.nih.gov/geo/

^2^ https://www.genecards.org/

^3^ https://cn.string-db.org/

^4^ http://117.50.127.228/CellMarker/

^5^ https://www.r-project.org/

^6^ https://posit.co/products/open-source/rstudio/
