# Supplementary material for: Exploring shared biomarkers and shared pathways in insomnia and atherosclerosis using integrated bioinformatics analysis
Source: Front Mol Neurosci. 2024 Oct 8;17:1477903. doi: 10.3389/fnmol.2024.1477903 (PMC11493776; doi:10.3389/fnmol.2024.1477903)
Supplement: Supplementary file 1 [file Data_Sheet_1.docx]

Supplementary Material

**1 Supplementary Tables**

**Table S1.** Detailed information about the datasets used in this study.

| Dataset | Database | Platform | Disease | Note |
| --- | --- | --- | --- | --- |
| Insomnia related genes | GeneCards |  | Insomnia | Test dataset |
| GSE100927 | GEO | GPL17077 | Atherosclerosis | Test dataset |
| GSE28829 | GEO | GPL570 | Atherosclerosis | Validation dataset |
| GSE208668 | GEO | GPL10904 | Insomnia | Validation dataset |
| GSE253903 | GEO | GPL24676 | Atherosclerosis | Test dataset |

**2 Supplementary Figures**


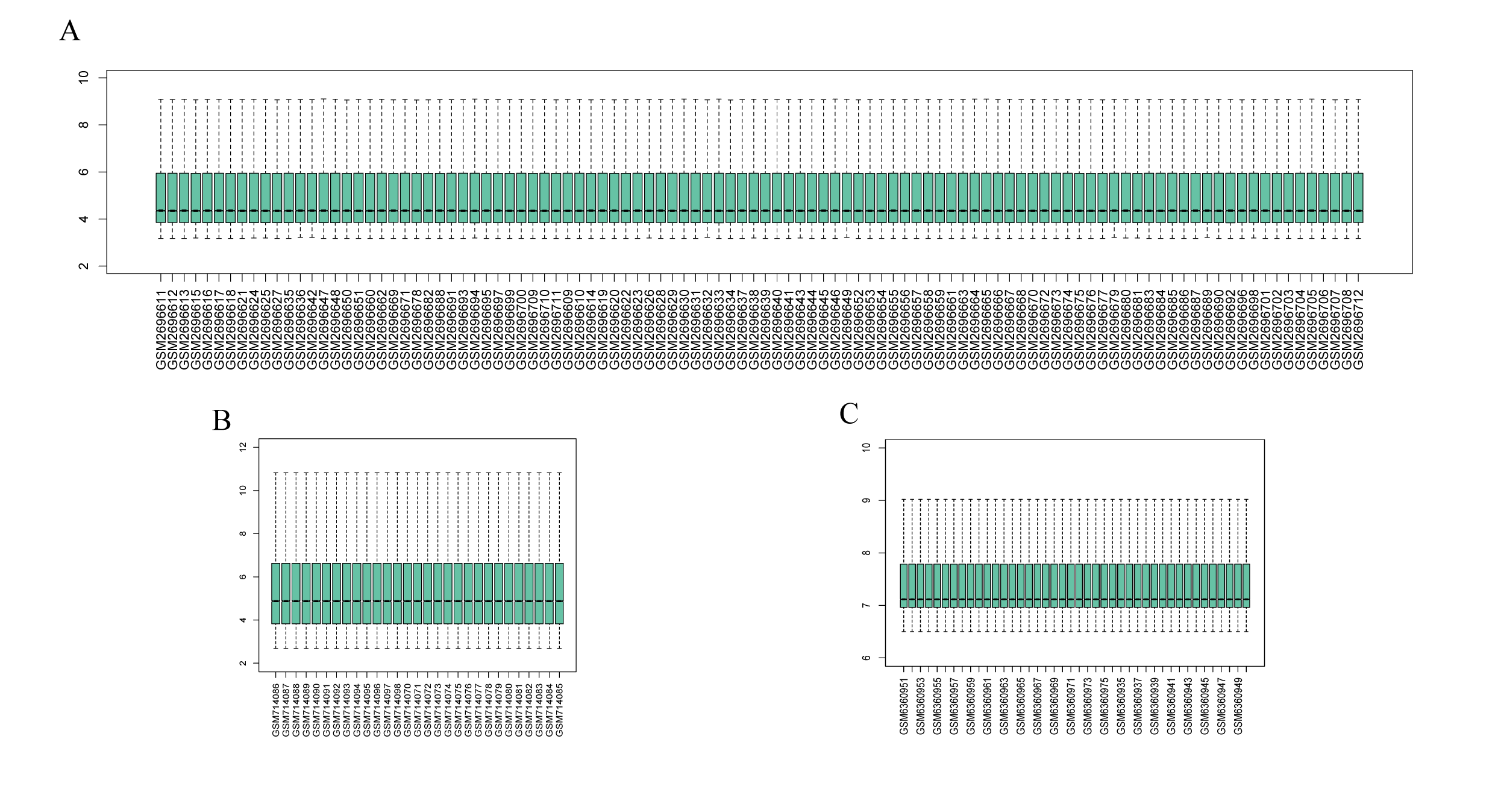


**Figure S1.** (A-C) The box plots of the data sets after removal of batch effects and normalization. A, GSE100927; B, GSE28829; C, GSE208668.


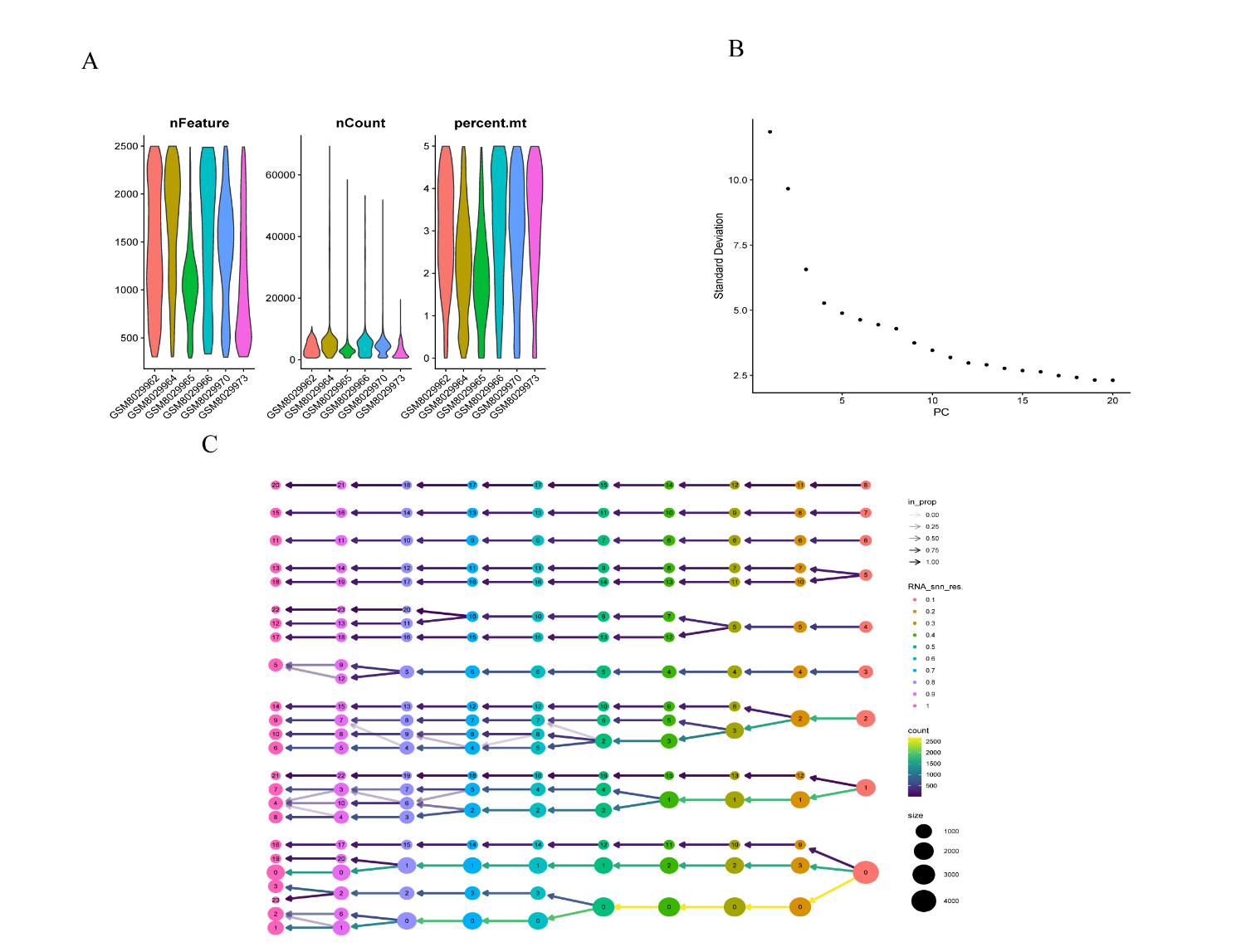


**Figure S2.** (A) The violin plot depicting the characteristics of the data after quality control in the GSE253903. nFeature, the number of genes detected per cell; nCount, the total expression per cell; percent_mt, the proportion of mitochondrial genes per cell. (B) Determination of principal component dimensions (dim). dim=1:20; PC, principal component. (C) Selection of clustering resolution. resolution=0.5.
